# Their “Jibbenenosey”

**Published:** 1882-02

**Authors:** 


### THEIR "JIBBENENOSEY."

Many years ago there lived on the then frontier, a man known among the white settlers as "Peaceful Nathan." The Shawnee Indians murdered his family, and that event made him no doubt insane and their unrelenting foe. To the white man he remained "Peaceful Nathan," as he carefully concealed from them his deeds of revenge. But to the Indians he was known as the "Jibbenenosey" (the devil). He killed scores of them secretly, and marked a cross with his knife on the heart of each one. For years he was the unknown terror of the savages, who whenever an Indian was missing, would be sure to find him lying dead in the forest, with the significant mark on his heart. "Jibbenenosey!" they would exclaim and flee from the spot.

When Milwaukee was a small town, one of its citizens, Colonel Clyman, was a Jibbenenosey to the Indians in the vicinity. Not one of them would remain in the town if he caught sight of the Colonel's face. The writer of the "Pioneer History" of the city relates that he once witnessed the effect of Clyman's presence upon a lot of Indians. They were lounging about a store when the Colonel entered. Instantly they started for the door, glancing behind, as if fearing an attack. In a moment the store was empty and the Indians out of sight.

Clyman's bitterness toward the savages arose from the fact that he had been nearly killed by them. He and a companion, named Burnett, started for Rock River in search of land. Purchasing a canoe from a squaw, they descended the river and put up at a deserted cabin.

The squaw's husband and son, coming home and learning what had become of the cenoe, started to kill the two white men. They wished to recover the canoe and to avenge the death of the squaw's brother, who had been killed by a white soldier.

The Indians entered the cabin, where Burnett was making a fire. The squaw's son shot him. The report of the gun and a screech of agony startled Clyman who was gathering wood in the forest. Looking up, he saw the old Indian standing in the doorway making signs for him to come up, as if Burnett had shot himself.

Clyman, suspecting no treachery, had nearly reached the cabin, when the Indian raised his gun to shoot him. He ran, jumping from side to side, to disturb the Indian's aim, who fired, breaking Clyman's left arm; the son also fired, shooting the Colonel's gun loaded with buckshot. The distance was already so far that the only effect of the shot was to slightly pepper Clyman's back. The Indians ran in pursuit. The Colonel hid under a fallen tree. So close was the search that the two Indians stood on the very tree and Clyman heard them talking. When they abandoned the chase, Clyman bound up his broken arm, and started through the wilderness for Milwaukee, fifty miles away.

Holding his broken left arm in his right hand, he traveled all that night amid a pouring rain. Without a morsel to eat, he went on through the next day and night, and reached Milwaukee in the forenoon of the second day, where his wounds were dressed.

The Indians were arrested, tried and sentenced to be hung, but the Governor of the territory pardoned them. The assassination, his own narrow escape, and the Governor's clemency made Clyman a bitter foe to the Indians, who feared him more than they did a regiment of soldiers. He was their "Jibbenenosey."

---

THE crowd gathered together on mill day at San Gabriel, Tex., were natives of many different States, and told jokes at the expense of Arkansas, "tar-heels" and others. One North Carolinian got after the half-dozen Arkansans hot and heavy. With other yarns he told the following: An emigrant preacher went into the Boston mountain region on a prospecting tour. Coming to a four-acre corn-patch, he fought his way through a dozen or more hounds and curs to a windowless cabin in its centre, and entering he commenced a conversation with the lady of the house by inquiring into the state of society thereabouts. The woman did not seem to understand his general inquiries, so he began to particularize: "What religion is most common around here?" Still she did not seem to understand. "Are there many Presbyterians around here?" he asked. "I don't know," she said. "My man John has hunted around here right smart for nigh on to sixteen years, and I don't reckon he's killed ary one." "Ah, madame!" said the good man, "I am afraid you live in darkness here." "Yes," she replied, glancing at the unbroken log walls, "yes, but John allows to cut out a winder next week." This was received with applause, and a true-blue Arkansan had the floor for reply. "I was traveling once in the old North State," he began, "and as I was riding across an opening like, I saw a man some little distance ahead of me, pointing, as I thought, a long gun at something up in a persimmon tree. I reined up my horse to wait for him to fire. After waiting some time and no firing done, I noticed the man did not seem to be taking any sight, but appeared to be shifting his piece from time to time, so I hailed him and asked him what he was up to. 'Raising pork for market,' answered he, without turning to me. I rode up, and that tar-heel had a little spotted shoat tied to a pole, holding it up to eat persimmons."—*Chicago News.*

